# Accelerated invagination of vacuoles as a stress response in chronically heat-stressed yeasts

**DOI:** 10.1038/s41598-018-20781-8

**Published:** 2018-02-08

**Authors:** Ayane Ishii, Masahito Kawai, Haruka Noda, Hiroyuki Kato, Kohei Takeda, Kotomi Asakawa, Yoshinobu Ichikawa, Tomohiro Sasanami, Keiji Tanaka, Yoko Kimura

**Affiliations:** 1 Graduate School of Integrated Science and Technology, Shizuoka University, Shizuoka, 422-8529 Japan; 2 Department of Agriculture, Shizuoka University, Shizuoka, 422-8529 Japan; 3 Laboratory of Protein Metabolism, Tokyo Metropolitan Institute of Medical Science, Setagaya-ku, Tokyo, 113-8613 Japan

## Abstract

When exposed to sublethal high temperatures, budding yeast cells can survive for a period of time; however, a sufficient amount of ubiquitin is necessary for this survival. To understand the nature of the stress, we examined the morphological changes in yeast cells, focusing on the vacuoles. Changes in vacuolar morphology were notable, and ruffled vacuolar membranes, accelerated invaginations of vacuolar membranes, and vesicle-like formations were observed. These changes occurred in the absence of Atg1, Atg9 or Ivy1 but appeared to require endosomal sorting proteins, such as Vps23, Vps24 or Pep12. Furthermore, the serial sections of the vacuoles analysed using an electron microscopic analysis revealed that spherical invaginated structures were linked together in a vacuole. Because degradation of cell surface proteins is induced from heat stress, fusion of endosomal and vacuolar membranes might occur frequently in heat-stressed cells, and yeast cells might be able to cope with a rapid increase in vacuolar surface area by such invaginations.

## Introduction

In response to elevated temperatures, organisms initiate a sequence of events that function to cushion these stresses^1–4^. In particular, the activities of protein quality-control systems, such as molecular chaperones or protein degradation machinery, are enhanced. In addition to the canonical heat shock response, the cell wall stress pathway and oxidative stress response are activated. Moreover, the transport systems, cytoskeletal organization and energy metabolism are also modulated.

These heat responses are commonly expected at elevated temperatures; however, there might be differences in the means of achieving thermotolerance, depending on the temperature and duration of heat. Molecular chaperone heat shock protein (Hsp)104, which is induced by brief heat stress such as 37 °C along with other heat shock proteins, is necessary for the thermotolerance at acute and lethal high temperatures, such as 50 °C for 10–20 min (induced thermotolerance)^5^. In contrast, the polyubiquitin-encoding gene *UBI4* is not necessary for the induced thermotolerance, but is required for chronic heat stress of sub-lethal high temperatures such as at 38.5 °C–41 °C (ref.^6^ and unpublished data)^6^. Under normal conditions e.g. at 25 °C, *UBI4* is neither expressed significantly nor required because the other ubiquitin-encoding genes *UBI1–3* provide enough of the ubiquitins needed for cell growth. Ubiquitin homeostasis is critical for the maintenance and growth of the cell, and the fact that *UB14* is induced under heat stress indicates that many ubiquitins are required for survival under such stress conditions^7^. Moreover, lysine(K)63-linked ubiquitination but not K48-linked ubiquitination, is critical for the survival of the heat stress^8^. Ubiquitination plays a variety roles in the cell; however, in general, K48-linked ubiquitination is utilized during proteasomal degradation, whereas K63-linked ubiquitination is utilized in events such as endocytosis and endosomal sorting toward vacuoles, suggesting that the latter functions are critical for survival during chronic heat stress^9^.

The reason why the different proteins are required to mitigate the two different types of heat stress has not been clearly elucidated. Because Hsp104 has been shown to disaggregate partially unfolded proteins^10,11^, it is considered that the protein unfolding damage is so severe at the lethal temperatures that disaggregation of the misfolded proteins, which are caused by the stress, would be the most critical point to rescue the cell. On the other hand, although the reason why cells need many ubiquitins after sublethal heat stress is not so clear, one can speculate that the protein-folding damage might not be severe enough to cause direct cell death. Rather, by removing unfolded proteins and using many ubiquitins, the cellular systems might be remodeled or reconstructed to adapt to such heat stress for the long-term survival of the cell. The report by Zhau that the toxic effects of overexpression of ubiquitin-substrates of cell surface proteins at higher temperatures supports this idea^12^. Indeed, the cell surface region appears to be remodeled after heat stress. For example, the degradation of several cell surface proteins, such as transporters, proton pumps or pheromone receptors, after various heat stresses, and the increase in chitin content, which is most likely activated by the cell wall stress pathway, have been reported^12–15^; however, the physiological changes or consequences that occur in the cell after chronic heat stress and that change the cell into a heat-tolerant state have not been fully investigated.

The vacuole is a degradative and dynamic organelle whose morphology changes in response to various stimuli or stresses^16,17^. Vacuoles fuse or fission depending on the stimuli: vacuoles fuse during stationary phase, starvation or under hypotonic conditions, and fission under hypertonic conditions. The autophagic responses are induced in response to nutrient starvation^18,19^. After heat stress, negative curvature formations of vacuolar membranes have been reported^20^. In addition, the vacuole is the place where several cellular surface proteins are degraded after heat stress.

To investigate the nature of the reactions to chronic sub-lethal heat stress, we started to look for the differences between wild type and *ubi4*Δ cells, and examine changes in cellular morphology in the yeast *Saccharomyces cerevisiae*. In this study, we focused on the morphological changes of the vacuoles after the stress, and further investigated any relevance to Δ*ubi4* and other mutations.

## Results

### Physiological properties under the chronic heat stress

**Figure 1:**
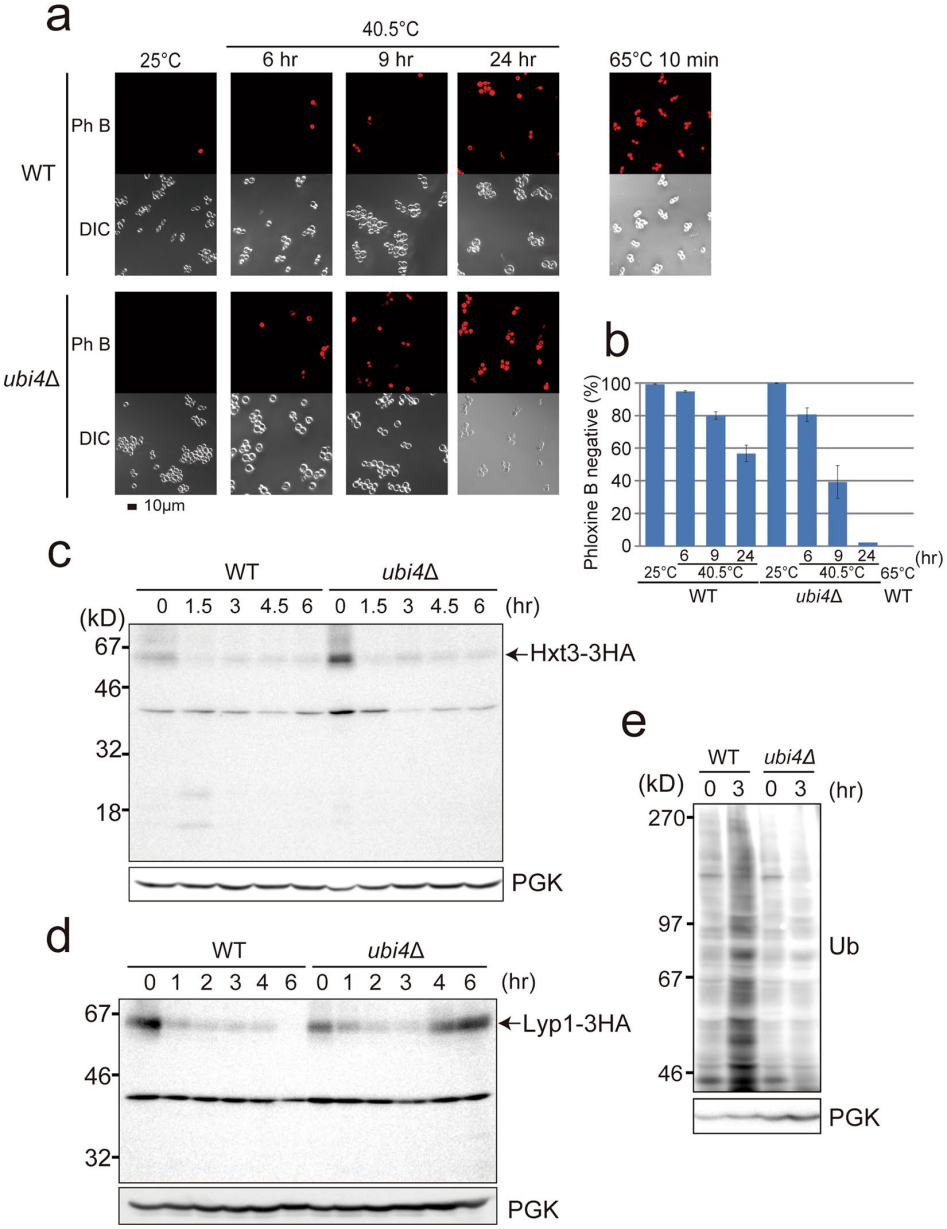
Physiological properties of cells under chronic heat stress in wild-type and *ubi4*Δ cells. (**a**) Phloxine B staining. Wild-type W303 and *ubi4*Δ cells grown at 25 °C were transferred to 40.5 °C, or wild-type cells incubated at 65 °C for 10 min and Phloxine B-stained cells were observed by fluorescence microscopy. Dead cells were stained by Phloxine B. (**b**) Quantification of Phloxine B-unstained cells in (**a**). Date are presented as the mean value of three independent experiments, except for the value of *ubi4*Δ cells at 40.5 °C for 24 h, which is an average of two experiments. Standard errors (SE) are shown. (**c**–**e**) Lyp1-3HA, Hxt3-3HA and ubiquitin accumulations in wild-type and *ubi4*Δ cells at the indicated times after exposure to 40.5 °C. Anti-HA antibody for Lyp1-3HA and Hxt3-3HA. Anti-phosphoglycerate kinase (PGK) antibody as a control for protein loading. Molecular weight markers are indicated. The levels of ubiquitinated proteins were lower in *ubi4*Δ cells than in wild-type cells after 3 h at 40.5 °C.

To better understand the effects of chronic sublethal heat stress, we first evaluated the viability at 40.5 °C, a sublethal temperature for yeast. Cell growth was retarded in wild-type cells after the shift to 40.5 °C, increasing by only 2.0-fold after 6 h compared with 13.4-fold and 7.9-fold at 25 °C and at 37 °C, respectively; however, staining the dead cells with Phloxine B revealed that 95% of the cells were alive after 6 h at 40.5 °C (Fig. 1a,b), but that this percentage decreased after 9 h, and reached 57% after 24 h. In the Δ*ubi4* mutant cells, the number of Phloxine B-stained cells sharply increased after 9 h, and nearly all cells were stained after 24 h.

After heat stress, several plasma membrane proteins, including Dip5, Ste3, Pma1, Can1, Hxt3, Mup1, Gap1, and Lyp1, have been reported to be ubiquitinated, transported and degraded in the vacuoles^12–14^. Moreover, overexpression of plasma membrane proteins have been shown to cause toxicity at elevated temperatures^12^. In accordance with these findings, Hxt3-3HA and Lyp1-3HA levels decreased in wild-type cells at 40.5 °C (Fig. 1c,d). However, in *ubi4*Δ mutant cells, Hxt3-3HA levels decreased, whereas Lyp1-3HA levels increased after an initial decrease, suggesting that the accumulation of the proteins that should be removed might be one of the reasons for the loss of viability in the *ubi4*Δ cells.

### Morphological changes in vacuoles after chronic heat stress

**Figure 2:**
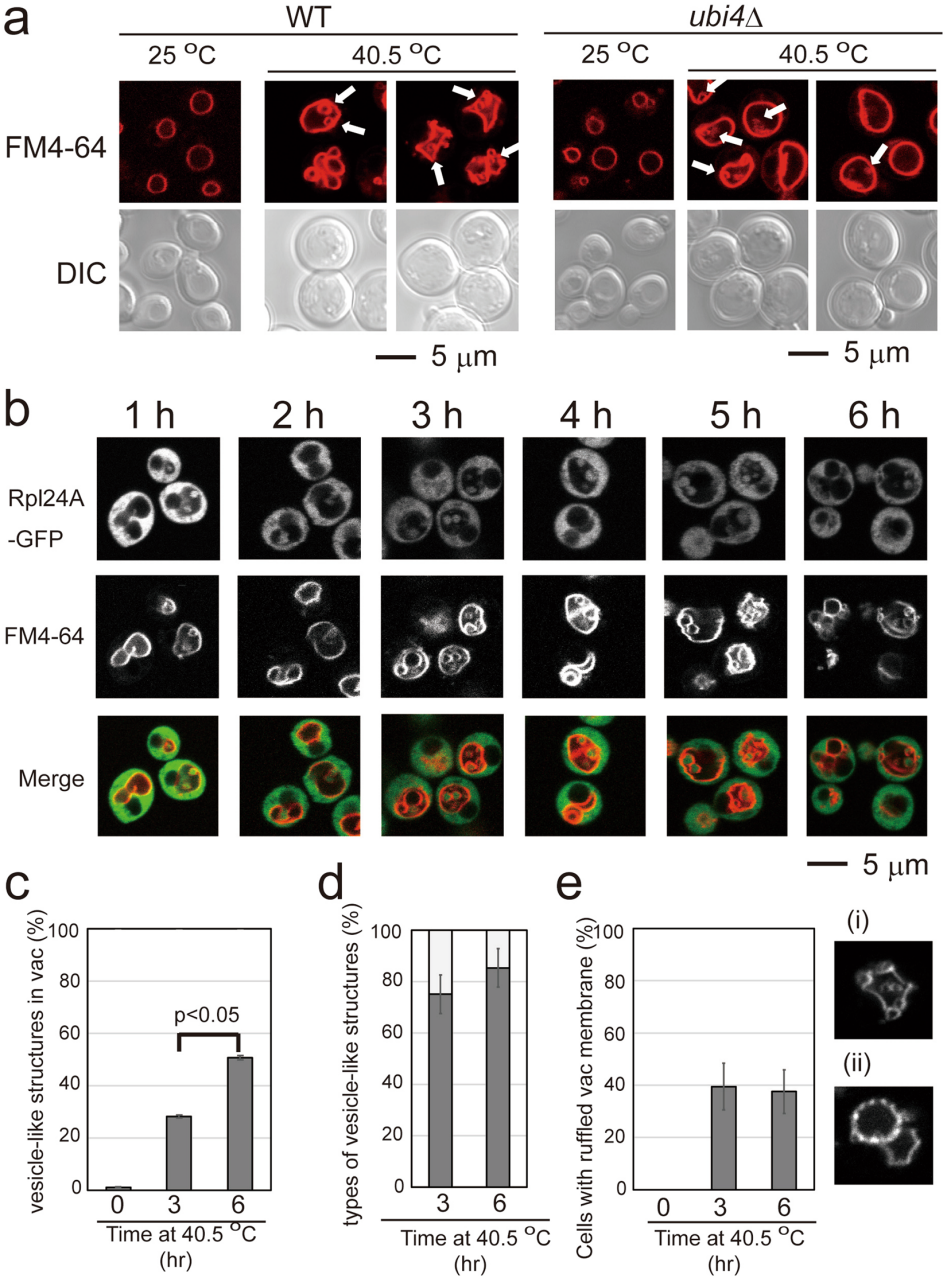
Changes in vacuolar morphology under chronic heat stress in wild-type and *ubi4*Δ cells. FM4-64 staining of wild-type W303 cells (left) and *ubi4*Δ cells (right) at 25 °C and 40.5 °C for 6 h. Two representative images are shown for cells at 40.5 °C. Arrows indicate vesicle-like structures. Scale bar, 5 μm. Because FM4-64 fluorescence was much darker in cells at 25 °C than in cells 40.5 °C for 6 h, the contrast of images of cells at 25 °C was enhanced. Images for the same contrasts at 25 °C and 40.5 °C are shown in Supplementary Fig. 1a. (**b**) Time course images of GFP and FM4-64 fluorescence of cells expressing Rpl24A-GFP at 25 °C and 40.5 °C. In each time, cells with vesicle-like structures in the vacuoles are shown. (**c**) Quantification of vesicle-like structures in the vacuoles. The ratios of cells which had vacuole(s) with vesicle-like structure(s). Experiments were performed six times. Error bar indicates SE, and statistical analysis was performed using Student’s t-test. (**d**) The ratio of vesicle-like structures with FM4-64 contour and GFP fluorescence inside is represented with a dark gray bar and vesicle-like structures with FM4-64 and GFP contours is represented with a light gray bar. Representatives of these structures are shown in Supplementary Fig. 1f. (**e**) The ratio of cells which had vacuole(s) with ruffled membranes. Vacuoles that looked like (**i**) and (**ii**) were counted. The results of (**d**) and (**e**) are the mean values of three independent experiments, and error bars indicate SE.

After a temperature shift to 40.5 °C, cells became larger and rounder (Fig. 2a). We decided to carefully examine the vacuolar structures in the cell after exposure to chronic heat stress because the protein transport toward the vacuoles involves the K63-linked ubiquitination, and the ruffling of vacuolar membranes by heat stress has been reported^20^. FM4-64 staining revealed that compared with the round and single-lobed structures of vacuoles at 25 °C, wild-type cells exhibited various vacuolar morphologies along with the fissions of vacuoles at 40.5 °C (Fig. 2a, Supplementary Fig. 1a). At 25 °C, 92% of cells contained only one vacuole and the rest contained more than two vacuoles, whereas after 6 h at 40.5 °C only 25% of cells contained only one vacuole. Vacuoles were highly distorted in some cells, such as negative membrane curvatures. In addition, we observed small vesicles in the vacuoles of some cells; some of which appeared to be anchored to vacuolar membranes, whereas others were mobile. Using a different strain, BY20695, we observed similar vesicles at 40.5 °C, but vacuole fission was not enhanced after heat stress. In this strain, 53% and 63% of the cells contained only one vacuole at 25 °C and 40.5 °C, respectively. The cells with vesicles were Phloxine B negative, indicating that they were viable (Supplementary Fig. 1c). It should be noted that the overall FM4-64 fluorescence on the vacuolar membrane was much brighter in cells at 40.5 °C than at 25 °C (Fig. 2a; Supplementary Fig. 1d,e) although the fluorescence was not always evenly distributed along the membrane at 40.5 °C (Fig. 2e(ii)), suggesting that FM4-64 uptake was accelerated at 40.5 °C.

To evaluate invaginations, we followed vacuolar morphological changes using yeasts that expressed Rpl24A-green fluorescent protein (GFP), a cytoplasmic ribosome subunit fused with GFP, following a temperature shift to 40.5 °C. Vesicle-like structures started to appear 1 h after the shift, although rarely, and increased by 6 h at 40.5 °C (Fig. 2b,c). We found that the majority of the vesicle-like structures exhibited GFP fluorescence surrounded by a FM4-64 fluorescent contour, and a portion of the vesicle-like structures was surrounded by both FM 4-64 and GFP fluorescence (Fig. 2d, Supplementary Fig. 1f). Ruffled vacuole membranes were increased after 3 h at 40.5 °C, and at a similar level after 6 h (Fig. 2e).

On examination of vacuolar structures in *ubi4*Δ cells, we observed that many vacuoles were swollen and remained single-lobed after exposure to heat stress (Fig. 2a, Supplementary Fig. 1a). At 25 °C, 89% of the cells contained only one vacuole, and at 40.5 °C, 77% of cells still contained one vacuole. Vacuole membranes were not significantly ruffled compared with those of the wild-type. Although vesicles were also detected within the vacuoles, these tended to be located near the vacuolar membranes, and extended vacuolar invaginations were not frequently observed compared with those in wild-type cells.

**Figure 3:**
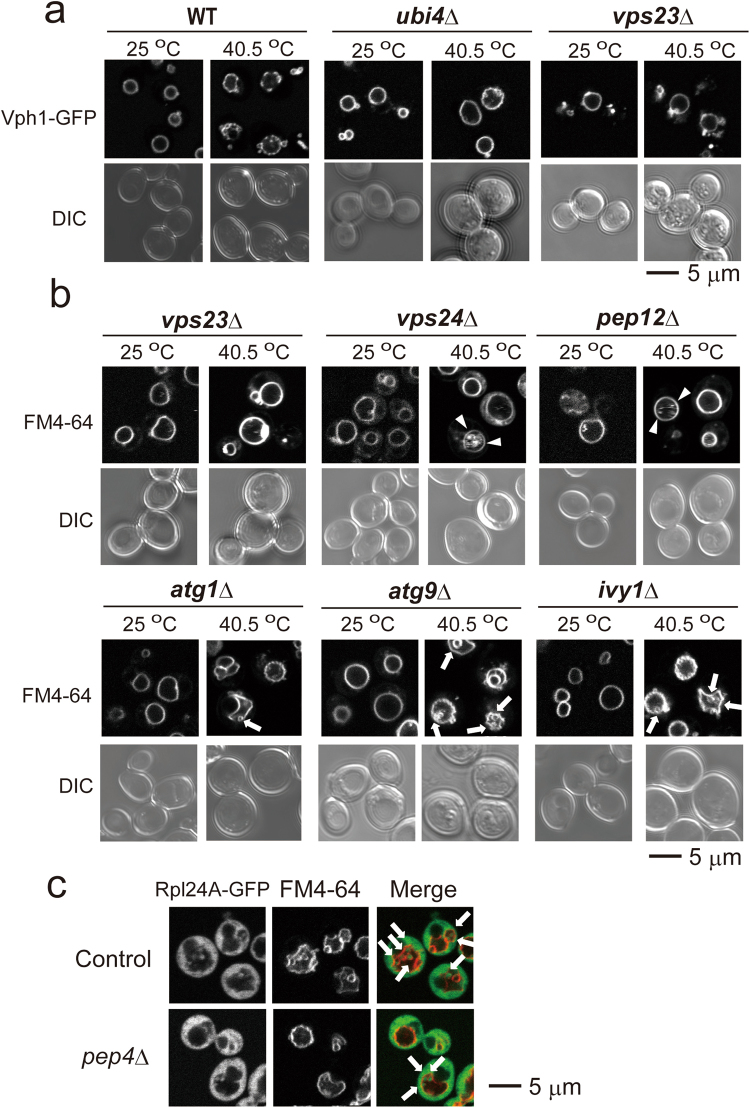
Changes in the vacuolar morphology under chronic heat stress in various mutant cells. (**a**) Vph1-GFP fluorescence of wild-type (ScHY-2445), *ubi4*Δ, and *vps23*Δ cells at 25 °C and 40.5 °C for 6 h. (**b**) FM4-64 staining of *vps23*Δ, *vps24*Δ, *pep12*Δ, *atg1*Δ, *atg9*Δ and *ivy1*Δ cells at 25 °C and 40.5 °C for 6 h. The contrast of images of cells at 25 °C was enhanced. Images for the same contrasts at 25 °C and 40.5 °C are shown in Supplementary Fig. 2. Arrows and arrow heads indicate vesicle-like structures and highly movable FM-4-64 fluorescent materials, respectively. (**c**) GFP and FM4-64 fluorescence of cells expressing Rpl24A-GFP at 40.5 °C for 6 h in control and *pep4*Δ cells. Arrows indicate vesicle-like structures.

Another method by which to follow the vacuolar membrane changes is to observe the localization of Vph1, a subunit of a vacuolar ATPase on the vacuolar membrane^21^. It has been reported that Vph1-GFP is rather homogeneously distribution on vacuolar membranes at normal temperatures but exhibits dotted localization after 3 h at 37 °C^22^. Consistent with that report, Vph1-GFP showed homogeneous distribution at 25 °C and dot-like localization after 6 h at 40.5 °C in wild-type cells (Fig. 3a). With Vph1-GFP fluorescence, vesicle-like structures were also observed. Interestingly, Vph1-GFP was uniformly distributed on vacuolar membranes at 40.5 °C in *ubi4*Δ cells. Because it has been suggested that the region that lacks Vph1-GFPis enriched with sterols in stationary-phase cells, there is a possibility that domains with different lipid contents are generated after the heat stress in wild-type cells, but not in *ubi4*Δ cells.

This observation of *ubi4*Δ cells was similar to the results of a previous report that some vesicular transport mutants exhibited a uniform distribution of Vph1-GFP at the stationary phase^22^; therefore, we considered Vph1-GFP fluorescence in the *vps23*Δ cells in which endosomal sorting was impaired. After heat stress at 40.5 °C, the mutant showed homogeneously distributed GFP fluorescence on the vacuolar membranes along with a foci structure, most likely the class E compartment, an aberrant late endosomal structure^23^. In addition, its vacuolar membrane was observed to be smooth, and budding-like structures of vacuolar membranes were not detected (Fig. 3a).

On the basis of these observations, we further examined the vacuole morphology of vesicular transport mutants with FM4-64 staining after chronic heat stress (Fig. 3b). We observed that *vps24*Δ, *vps23*Δ and *pep12*Δ mutants exhibited similar phenotypic vacuolar morphology; their vacuolar membranes did not show significant invaginated structures and remained smooth after chronic heat stress. In these cells, highly mobile FM4-64 fluorescent materials, which did not look like vesicle structures, were often observed in their vacuoles. These mutants were reported to be heat-sensitive^24,25^. Indeed, some of the *vps23*Δ and *vps24*Δ cells appeared to be dying after 6 h at 40.5 °C (data not shown), and they showed loss of viability after the chronic heat stress (Supplementary Fig. 3).

To determine whether the vesicle-like structures were generated from autophagic events, we examined the effects of *atg1*Δ and *atg9*Δ mutations (Fig. 3b). Both Atg1 and Atg9 are required for macroautophagy^26^. The vacuolar morphologies of these mutants after 6 h at 40.5 °C were very similar to those of the wild type. Next, we examined *ivy1*Δ mutations after chronic heat stress, because Ivy1 was reported to be localized at the invaginated vacuolar membranes and implicated in microautophagy, an inward budding of the cytoplasm from the vacuolar membrane^20,27^. Consistent with previous reports on invaginations after heat stress in the *ivy1*Δ mutant^20^, we observed both the invaginations and the vesicle-like formations in the mutant after chronic heat stress (Fig. 3b). These results suggest that the formation of vesicle-like structures after chronic heat stress does not involve either the macroautophagy process or Ivy1. In addition, increases in vesicle-like structures were not observed in the *pep4*Δ mutant after 6 h at 40.5 °C (Fig. 3c), which suggested that these structures were apparently not degraded by Pep4.

**Figure 4:**
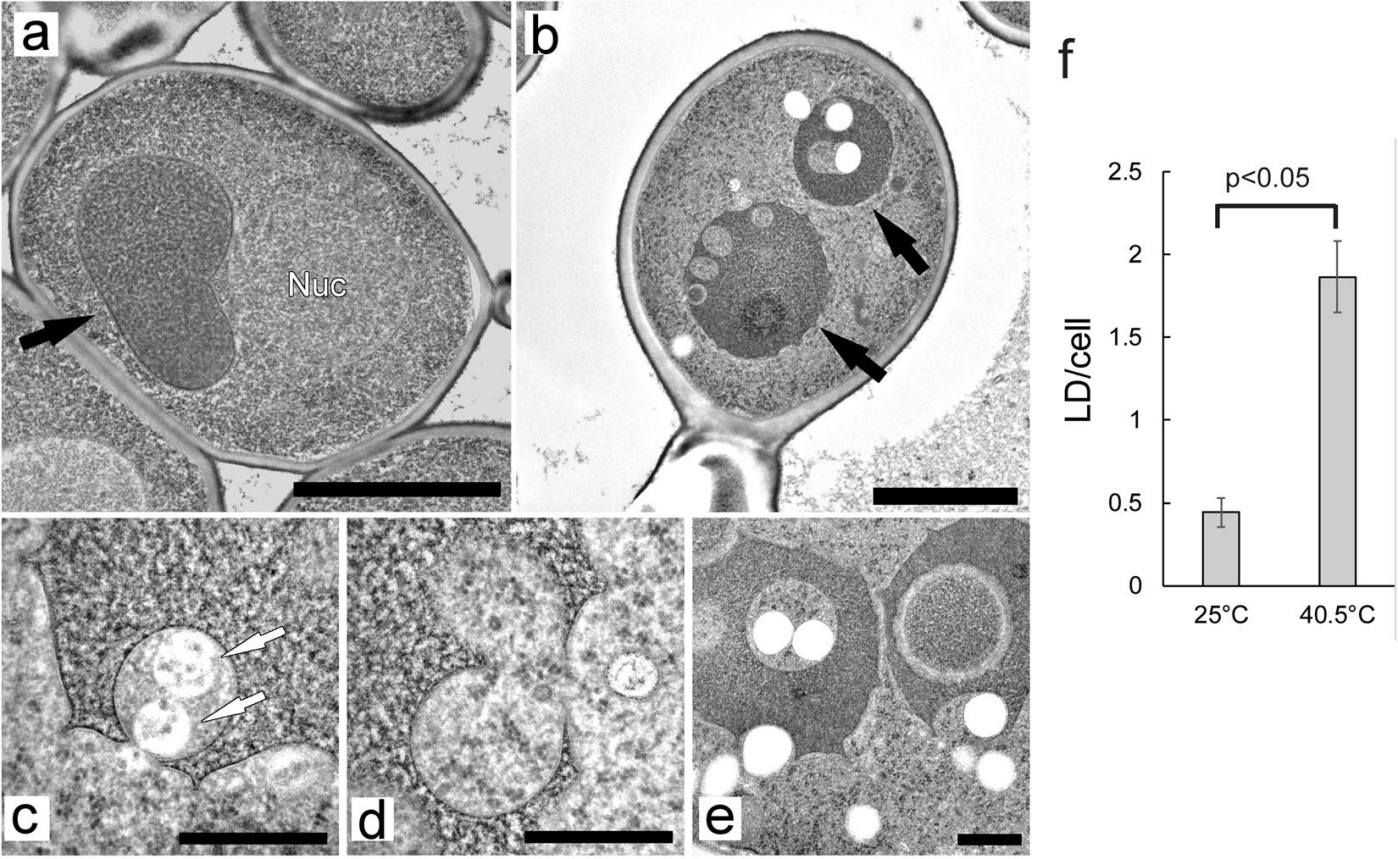
Electron microscopy of wild-type cells, and lipid droplet formation after chronic heat stress. (**a**) Wild-type cells at 25 °C. (**b**) Wild-type cells grown at 40.5 °C for 6 h. (**c**–**e**) Various types of vacuolar invaginations in cells grown at 40.5 °C for 6 h. Scale bar; 2 μm for (**a**) and (**b**), and 0.5 μm for (**c**–**e**). Black and white arrows indicate vacuoles and MVBs, respectively. (**f**) Number of lipid droplets per a cell at 25 °C and 40.5 °C for 6 h. Average were calculated from EM images of fifty-two cells that were randomly taken. Error bar indicates SE, and significant difference was calculate using Student’s-t test.

We then examined the fine structures using electron microscopy (EM). The vacuoles exhibited smooth membranes in wild-type cells at 25 °C (Fig. 4a). In contrast, vacuoles of cells at 40.5 °C exhibited various shapes (Fig. 4b–e, Supplementary Fig. 4). Vacuolar membranes were angular or not smooth in about half of the cells. At a frequency similar to the light microscopic analysis, the invagination of the cytoplasm and vesicle-like structures were observed in EM. Multiple vesicular bodies (MVBs) were occasionally observed in bud-like invaginations (Fig. 4c). Invaginated structures with a bumpy morphology were also occasionally observed (Fig. 4d). In addition, doughnut-like structures in which a belt of cytoplasm surrounded the vacuolar lumen were occasionally observed (Fig. 4b,e). These might be the vesicle-like structures surrounded by GFP and FM4-64 fluorescence in cells expressing Rpl24a-GFP as shown in Fig. 2d and Supplementary Fig. 1f; however, how these structures were formed remains unclear. During EM analysis, we unexpectedly noticed more lipid droplets in cells after 6 h at 40.5 °C than at 25 °C (Fig. 4b,e,f). In addition, these were often observed at the invaginated vacuolar membrane or in contact with vacuolar membranes.

**Figure 5:**
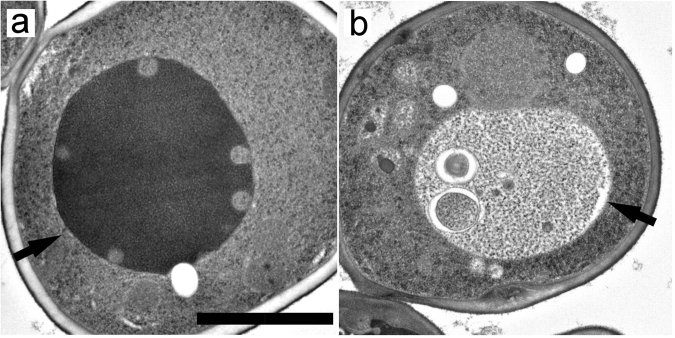
Electron microscopy of *ubi4*Δ, and *vps2*3Δ cells grown at 40.5 °C for 6 h. (**a**) *ubi4*Δ cells. (**b**) *vps2*3Δ cells. Scale bar; 2 μm. Arrows indicate vacuoles.

In *ubi4*Δ cells, we also observed bud-like invaginations after 6 h at 40.5 °C, although these were tended to be located near the vacuolar membranes, and vacuolar membranes were smoother than those in wild-type cells (Fig. 5a, Supplementary Fig. 5), which was consistent with the images seen in FM4-64 staining. Vacuolar membranes in *vps23*Δ cells were much smoother, and vesicle-like cytosolic structures were not observed after 6 h at 40.5 °C (Fig. 5b, Supplementary Fig. 6). Instead, ~40% of the *vps23*Δ cells contained unidentified constituents within the vacuoles; these might be the FM4-64 fluorescent highly mobile constituents shown in the vacuole in Fig. 3b.

**Figure 6:**
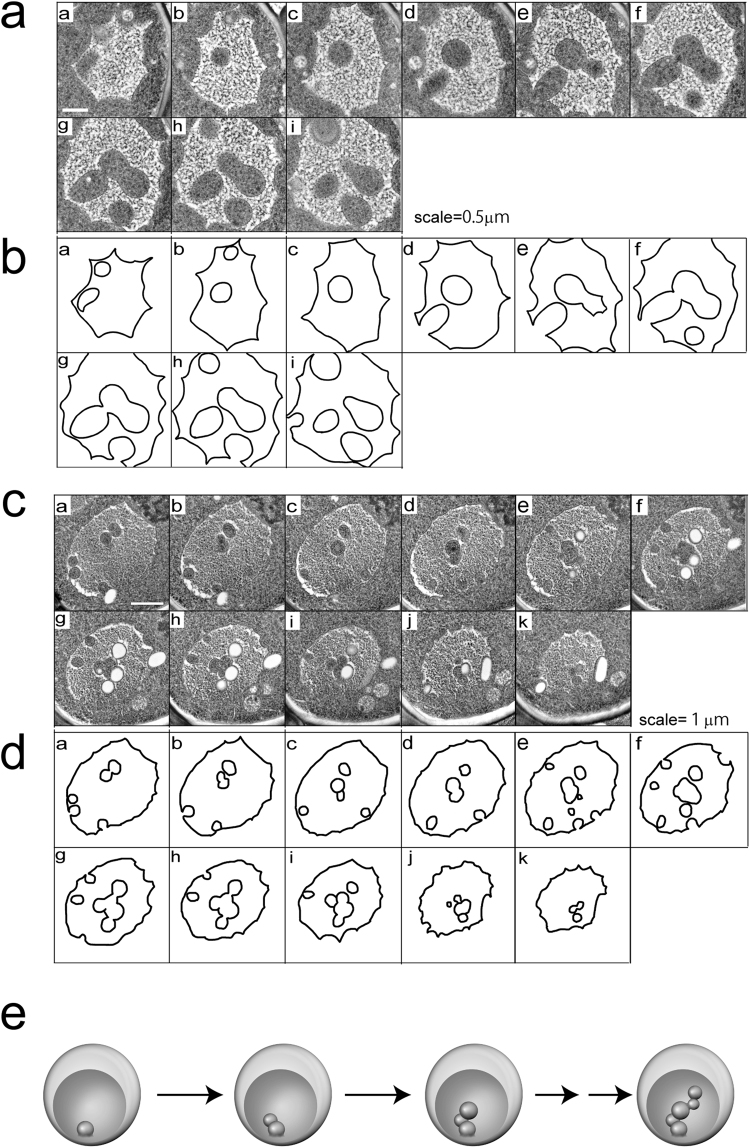
Serial sections of vacuoles. (**a**) and (**c**) Electron micrographs of 80-nm-thin serial sections of a part of a vacuole in wild-type cells grown at 40.5 °C for 6 h. (**b**) and (**d**) Traces of vacuolar membranes of (**a**) and (**c**), respectively. Scale bar; 0.5 μm for (**a**) and 1 μm for (**c**). (**e**) Model for the formation of invaginations in vacuoles in chronically heat-stressed cells.

Finally, three-dimensional observations (3D) of serial sections of vacuoles were conducted to analyse the vesicle-like structures (Fig. 6; Supplementary Fig. 7; Supplementary Videos 1,2,3,4,5 and 6). Surprisingly, vesicle-like structures did not exist independently; instead, several vesicle-like structures were either linked together in a vacuole or connected to the cytoplasm. The average diameter of the vesicle-like structures was calculated to be ~0.6 μm using the images of the serial sections. Sometimes, we observed lipid droplets within the linked structures. Among the six series of serial sections observed, we did not find any free spherical vesicles. Although the possibility of microautophagy for the vesicle-like structures remained, it was suggested that many vesicle-like structures observed in the vacuoles did not form a free single sphere in chronically heat-stressed cells.

## Discussion

To understand the nature of chronic sublethal heat stress on yeast, we examined the physiological changes in the cells after the stress, and focused on the specific changes in vacuolar morphology in this study. We observed vesicle-like structures that formed in vacuoles when subjected to the chronic heat stress, and observations of serial sections of the vacuoles using EM revealed that these vesicle-like structures were linked. In addition, our results suggest that endosomal sorting proteins Vps23, Vps24 and Pep12 are necessary for invagination of vacuolar membranes and vesicle-like formation after heat stress.

We suggest that linked vesicle-like structures are a new type of vacuolar invaginated structures observed in chronically heat-stressed cells. Together with our results that showed these endosomal sorting proteins are needed, we propose a model that explains the formation of these structures (Fig. 6e). After heat stress, several endosomes fuse with vacuolar membranes, and bud-like invaginations are formed because of the rapid increase in these membranes. It could be speculated that the number of endosomes that fuse with the vacuoles might increase after heat stress because more cell surface proteins were reported to be lost as the temperature increased^12^. The endosomes travel to these invaginations, forming new invaginations from existing ones. The subsequent endosomes are formed in the same manner, and result in chains of vesicle-like structures. If our speculation is correct, the cells might be able to cope with massive increases in vacuolar membranes by forming such invaginated structures without an increase in vacuolar volume; however, to test this model and to determine the process by which the linked vesicle-like structures are formed, time-lapse analysis is needed to capture the onset of invagination.

In addition, additional studies are needed to determine whether the formation of these linked structures is a reversible process. A preliminary study demonstrated that when the temperature was returned to 25 °C following exposure to a temperature of 40.5 °C for 4 h, a decrease of vesicle-like structures occurred in the vacuole after 2 h; however, when the temperature was returned to 25 °C after 40.5 °C for 6 h, this significant decrease was not evident after 2 h (data not shown). Thus, we speculate that invaginations are easily reversible in the early stages, and that excessive invaginations are irreversible or take time to revert.

Vesicle-like structures in vacuoles have been reported previously^27,28^. For example, vesicle-like structures in the vacuole were reported by the addition of glycerol^28^. We are not certain whether these are the same vesicle-like structures; such structures could be formed not only because of chronic heat stress but also because of other environmental changes. In addition, vesicle-like structures might be intralumenal fragments, which are produced during vacuolar fusions^29^. However, in the W303 strains used in this study, vacuoles were rather frequently fragmented by heat stress, therefore, we do not think this is likely. Moreover, from the results of Vph1-GFP distribution, we suggest that the raft-like domains having different sterol contents, which could be formed in the vacuolar membranes under heat stress, might affect the invagination region and frequency in forming vesicle-like structures.

In our study, the *ubi4*Δ mutant exhibited less clear phenotypes in terms of vacuolar structures. The vesicle-like structures in the vacuole were observed in the mutant like as wild-type, but the vacuolar membranes of the *ubi4*Δ mutant were smoother than those of the wild type, but less smooth than those of the *vps23*Δ mutant. Regarding the Vph1-GFP fluorescence, both *ubi4*Δ and *vps23*Δ mutants exhibited homogeneous distribution, whereas wild-type cells exhibited dotted localizations on the vacuolar membrane at 40.5 °C. We speculate that the impairment of ubiquitination in *ubi4*Δ cells might result in decreased transport of endosomes to vacuoles and fewer fusion events of vacuolar membranes and endosomes, resulting in the observed vacuolar structures and membranes.

The invagination of the vacuoles and the vesicle-like formations in the vacuoles occurred in the absence of either Atg1 or Atg9, which are essential for macroautophagy, and of Ivy1, which has been implicated in microautophagy; therefore, the phenomena of macroautophagy and microautophagy, which are dependent on these Atg proteins and Ivy1, are not involved in this process. Although the increase in vesicle-like structures containing Rpl24A-GFP was not detected in *pep4*Δ cells after 6 h at 40.5 °C, whether microautophagy occurs after chronic heat stress has yet to be investigated. Microautophagy that was dependent on endosomal sorting proteins but independent of core Atg protein(s) was recently reported to occur after a diauxic shift or a chronic phospholipid deficient state^30,31^. Similarly, the degradation of an ubiquitinated vacuolar membrane protein in the vacuoles has shown to require endosomal sorting proteins^32^. Therefore, it is reasonable to think that similar events might occur after chronic heat stress.

During the course of EM study, we unexpectedly found that more lipid droplets were produced after a shift to 40.5 °C for 6 h. We found that lipid droplets were present in the invaginations, and, from EM images of serial sections of the vacuoles, that some lipid droplets were linked to cytoplasmic vesicle-like structures. The physiological implication of lipid droplet localization around the vacuoles has not been investigated, but it is possible that lipid droplets are sequestered in the invaginated area of the vacuoles during heat stress. The lipid droplets produced under several different conditions were reported to be digested, possibly through microautophagy^31,33–36^. Thus, we are currently investigating whether microlipophagy occurs under chronic heat stress.

Finally, due to the progress of global warming, it is likely that more and more organisms are expected to have a chance to be exposed to sub-lethal temperatures in the future^37^. We believe that our study will contribute to the understanding how the cellular activities are influenced by these temperatures.

## Methods

### Media and yeast strains

Yeast strains were grown in YPAD medium [1% yeast extract, 2% Bacto–Peptone or Hipolypepton (Nihon Seiyaku), 2% glucose, and 0.002% adenine]. A list of yeast strains is provided in Supplementary Table 1. W303 strains were used if not indicated. Vph1-GFP-expressing-yeasts and *pep12*Δ mutants were SEY6210 and BY4741 strains, respectively.

### Determination of cell viability

For yeast viability assays, yeast cells at the early log phase grown at 25 °C were transferred to 40.5 °C, and incubated with a moderately slow shaking speed (70 turns/min) in a water temperature-chamber. Staining with Phloxine B was performed using the modified method of Noda^38^. Briefly, cells were pelleted by centrifugation at 300 × g for 3 min and washed twice with 1x PBS. This was followed by resuspension in 1x PBS containing Phloxine B at a final concentration of 5 μg/ml. Cells were observed under a fluorescence microscope using a red filter. In total, approximately 200 cells were observed after each staining, and the ratio of Phloxine B-negative cells to the total number of cells was calculated.

For the colony formation assay, 10 μl of culture was diluted in 10 ml of 1x PBS, plated onto YPAD medium in duplicates, and incubated at 25 °C for 2 days. The culture was directly plated onto the YPAD medium when the cell viability was very low.

### Immunoblotting

Preparation of whole-cell extracts and immunoblot analysis were performed essentially as described previously^39^. Cells (1–3 × 10^7^) were washed with water and suspended in 200 μl of cold ethanol containing 2 mM PMSF. Cells were broken by agitation with 200 μl of glass beads for 10 min, and chilled at −20 °C. Cells were dried, suspended in sample buffer and heated at 96 °C for 5 min. In western blotting, the blots were incubated with mouse anti-GFP monoclonal antibody (Roche), anti-HA antibody (TANA2, MBL), anti-ubiquitin antibody (P4D1, Santa Cruz), or anti-yeast phosphoglycerate kinase (PGK) antibody (Molecular Probes, Eugene, OR), followed by horseradish peroxidase (HRP)-conjugated anti-mouse IgG (#NA931V, GE Healthcare), and visualized using a chemiluminescent reagent.

### Microscopy

FM4-64 staining was performed as described previously^40^, and the cells were treated with FM 4–64 just before the temperature shift. For the treatment of FM4-64, 2 ml culture of cells grown at 25 °C in YPAD medium were centrifuged at 5000 rpm for 1 min, and suspended in 49 μl of YPAD. To the cells, 1 μl of 2 mM FM4-64 (Molecular Probes, Inc.) was added at a final concentration of 40 μM, and incubated for 20 min at room temperature. Then cells were washed with 1x PBS and suspended in 2 ml of YPAD, followed by the heat treatment. After heat treatment, cells were collected by centrifugation and imaged at room temperature using a confocal microscope (LSM700; Carl Zeiss) equipped with a 100x oil or 40x water objective lens. Microscopic observation started 10–15 min after the heat treatment. Images were processed using the LSM image browser, and the brightness and contrast were adjusted using Zen.

### Electron microscopy

Electron microscopy was performed by Tokai-EMA, Inc. Rapid freezing and freeze-fixation methods were employed. After collection of cells by centrifugation, the small number of cells were sandwiched between copper disks, and frozen in liquid propane at −175 °C. They were freeze-substituted with 2% glutaraldehyde and 0.5% tannic acid in acetone, followed by 2% distilled water at −80 °C for 2 days. The cells were fixed with 2% osmium tetroxide in acetone. The cells were then dehydrated, infiltrated with propylene oxide, embedded in Quetol-651 and observed using a transmission electron microscope (JEM-1400Plus; JEOL Ltd.). Serial sections were sliced at approximately 80 nm. Images were adjusted using GIMP. 3-D reconstructions of EM images were performed by using Image J software. Flip EM images were assembled using GIMP.

### Data availability

No datasets were generated or analyzed during the current study.

## Electronic supplementary material

Supplementary figures and table

Supplementary Video 1

Supplementary Video 2

Supplementary Video 3

Supplementary Video 4

Supplementary Video 5

Supplementary Video 6
