# Supplementary material for: Accelerated invagination of vacuoles as a stress response in chronically heat-stressed yeasts
Source: Sci Rep. 2018 Feb 8;8:2644. doi: 10.1038/s41598-018-20781-8 (PMC5805771; doi:10.1038/s41598-018-20781-8)
Supplement: Supplementary file 1 — Supplementary figures and table [file 41598_2018_20781_MOESM1_ESM.pdf]

Supplementary methods, figures and table

Accelerated invagination of vacuoles as a stress response in chronically heat-stressed yeasts

Ayane Ishii[a], Masahito Kawai[a], Haruka Noda[a], Hiroyuki Kato[b], Kohei Takeda[a], Kotomi Asakawa[b], Yoshinobu Ichikawa[b], Tomohiro Sasanami[a], Keiji Tanaka[c] and Yoko Kimura[a,b,#]

[a]Graduate School of Integrated Science and Technology, Shizuoka University, Shizuoka, 422-8529, Japan.
[b]Department of Agriculture, Shizuoka University, Shizuoka, 422-8529, Japan.
[c]Laboratory of Protein Metabolism, Tokyo Metropolitan Institute of Medical Science, Setagaya-ku, Tokyo, 113-8613, Japan.

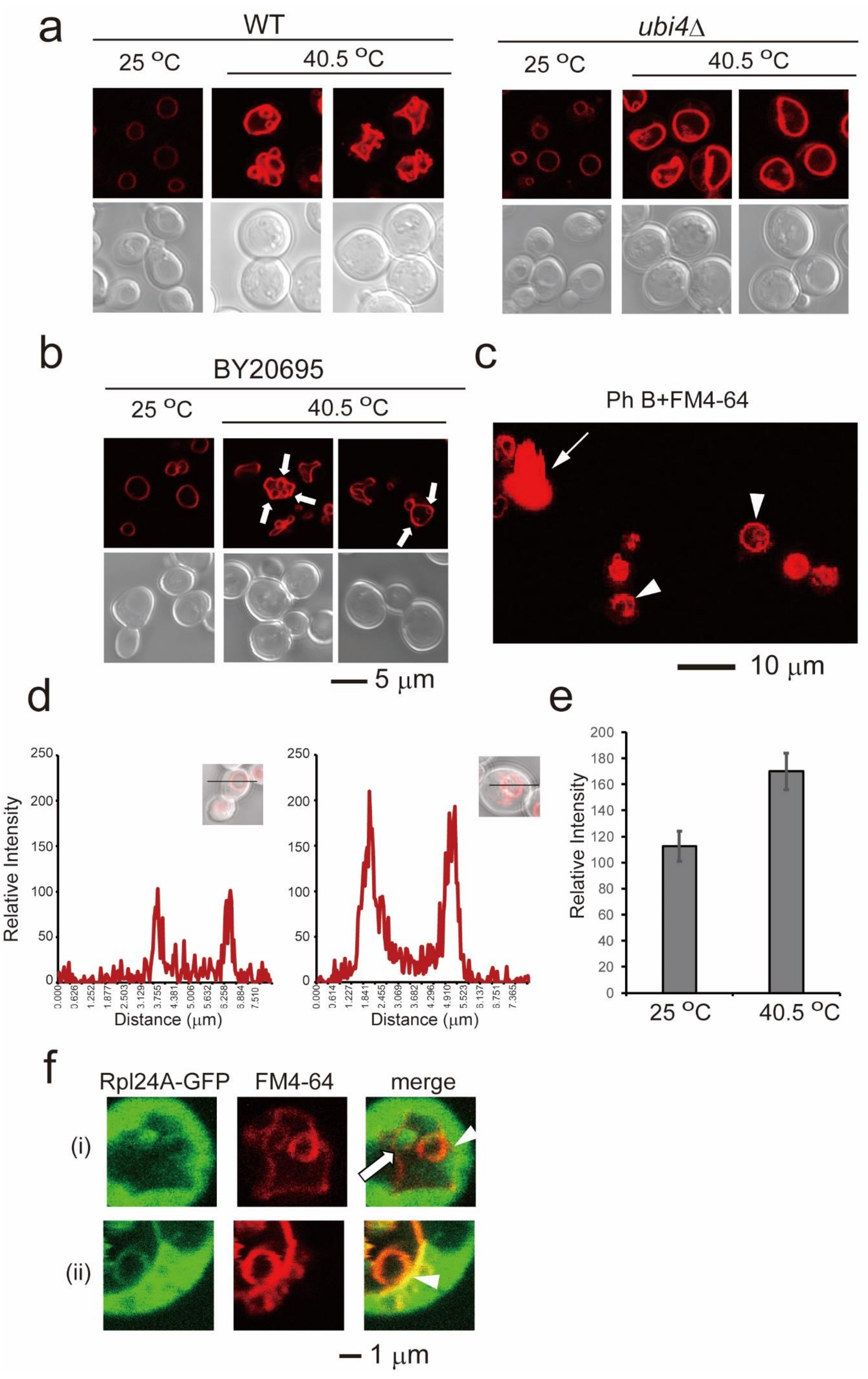
a
WT
25 °C
40.5 °C
ubi4Δ
25 °C
40.5 °C
b
BY20695
25 °C
40.5 °C
5 μm
c
Ph B+FM4-64
10 μm
d
Relative Intensity
Distance (μm)
e
Relative Intensity
25 °C
40.5 °C
f
Rpl24A-GFP
FM4-64
merge
(i)
(ii)
1 μm

2

Supplementary Figure 1. FM4-64 staining

(a). FM4-64 stained cells with equal degree of contrast adjustments of images between the cells grown at 25°C and 40.5°C for 6 h in Figure 2a. (b) FM4-64 staining of strain BY20695 cells grown at 25°C and 40.5°C for 6 h. Arrows indicate vesicle-like structures. (c). FM4-64 and Phloxine B double staining of wild-type cells grown at 40.5°C for 6 h. The arrow indicates both Phloxine-B and FM4-64 stained cells, and the arrowheads indicate FM4-64 stained cells with vesicle-like structures. Scale bar, 10 μm. (d). Line scan of FM4-64 fluorescence across the wild-type cells at 25°C (left) and 40.5°C for 6 h (right). Representative scan profiles are shown. (e) Quantification of FM4-64 in (d). Mean value of a max peak intensity of ten vacuolar membranes and error bar (SE) is shown. (f). (From Figure 1d). Representatives of vesicle-like structures with FM4-64 contour and GFP fluorescence inside, and structures with both FM4-64 and GFP contours, and they are indicated by arrow and arrowhead, respectively.

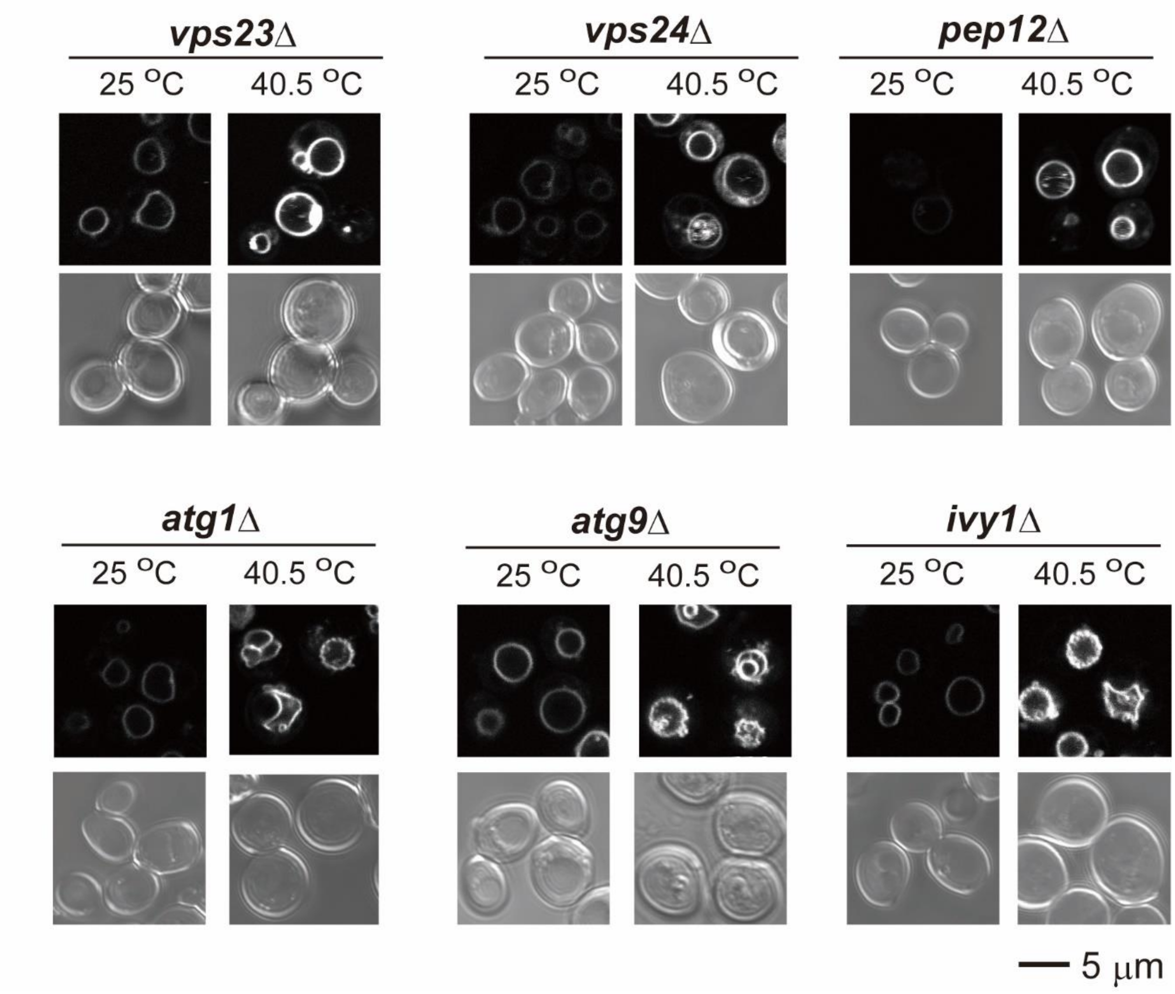

Supplementary Figure 2.

FM4-64 stained cells of various mutants with equal contrasts

FM4-64 stained cells of various mutants with equal contrasts for cells grown at 25°C and 40.5°C in Fig. 3b.

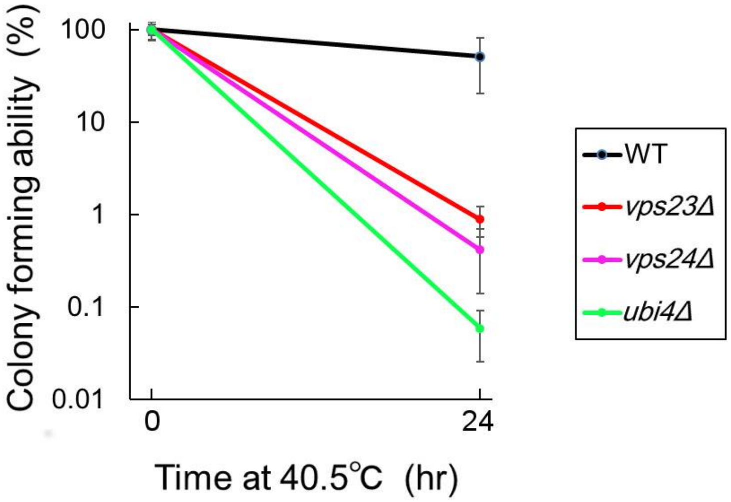

Supplementary Figure 3.

Colony forming abilities

Colony forming abilities of wild-type, Δ*ubi4*, Δ*vps2*3 and Δ*vps24* cells grown at 40.5°C for 24 h. Percentage viabilities by comparing the number at the starting time are shown. Results are mean value of three independent experiments. Error bar indicates SE. The viability of *pep12*Δ cells was not examined.

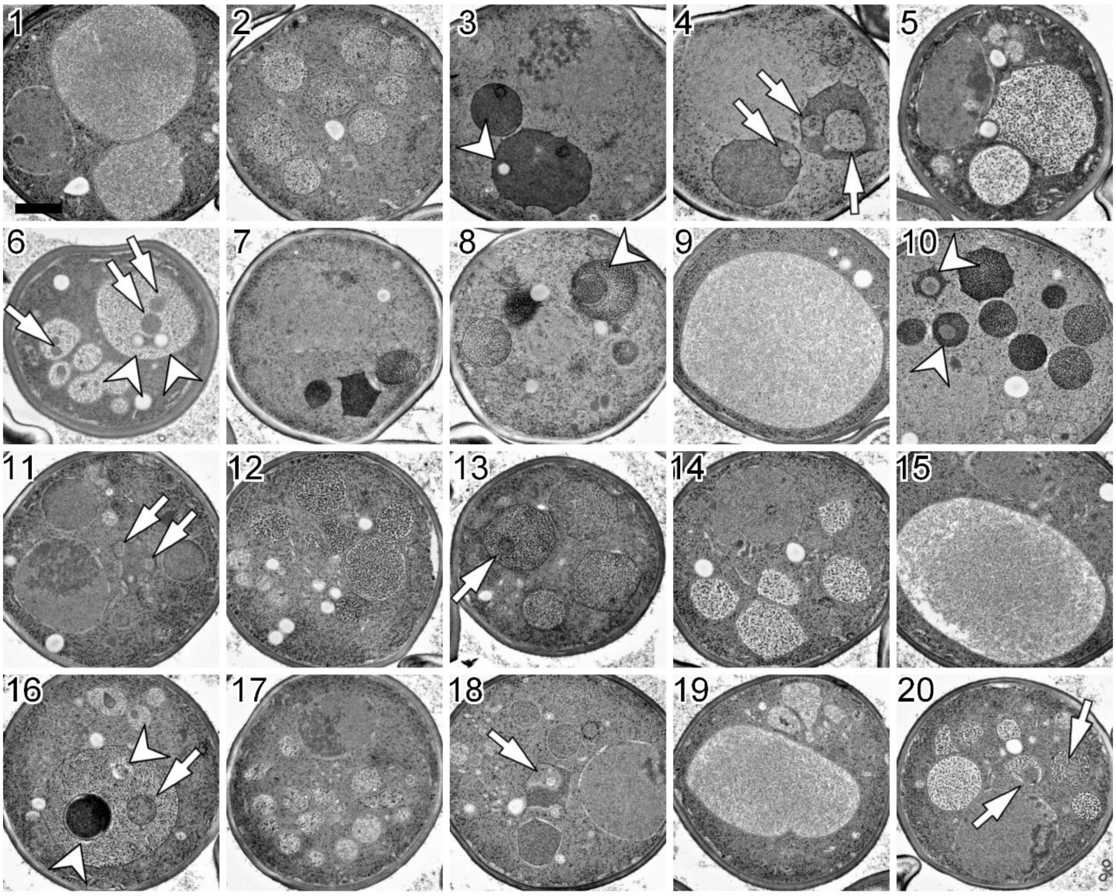

Supplementary Figure 4.

Electron microscopic analysis of wild-type cells grown at 40.5°C for 6 h

Fifty-two images were randomly taken, and of these, twenty representative cells to show the overall population, are shown. Arrows and arrow-heads indicate cytoplasmic-like and non-cytoplasmic vesicle-like structures, respectively, as judged form the existence of ribosome-like structures. Scale bar, 1 μm.

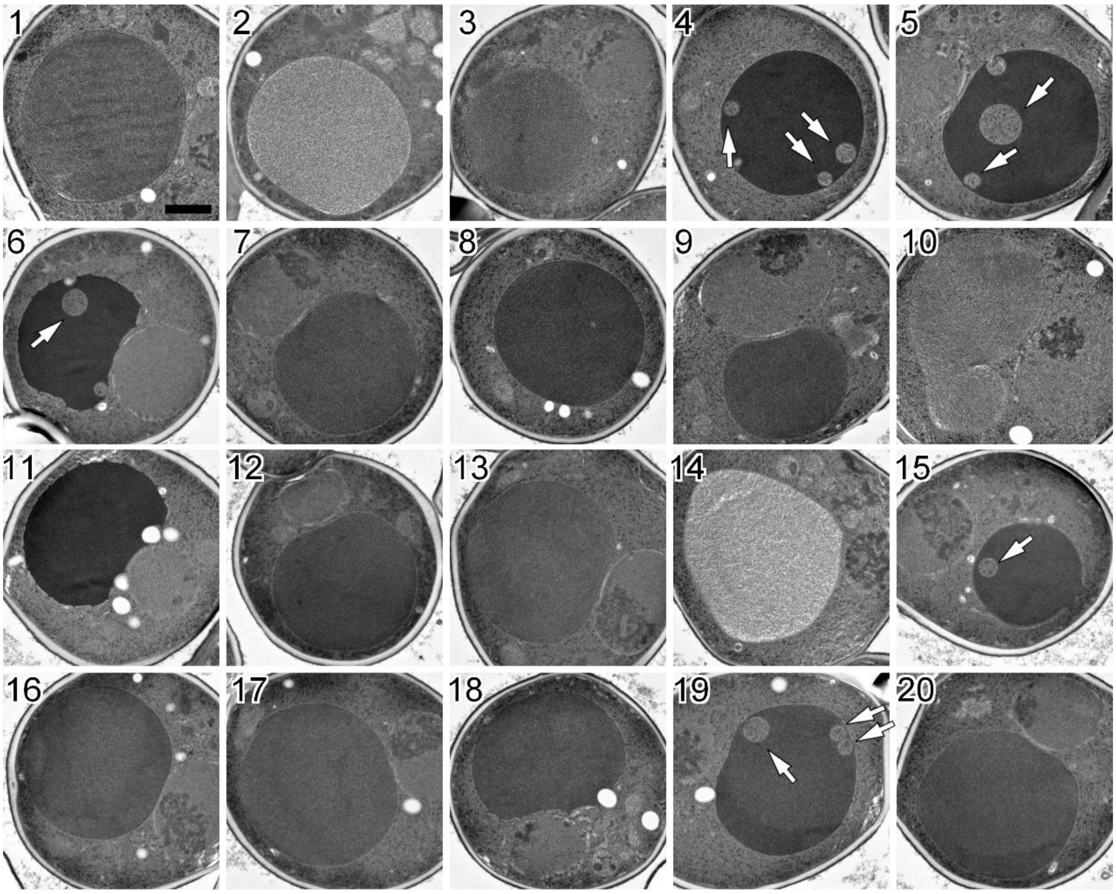

Supplementary Figure 5.

Electron microscopic analysis of *ubi4*Δ cells grown at 40.5°C for 6 h

Fifty images were randomly taken, and of these, twenty representative cells to show the overall population, are shown. Arrows indicate cytoplasmic vesicle-like structures. Scale bar, 1 μm.

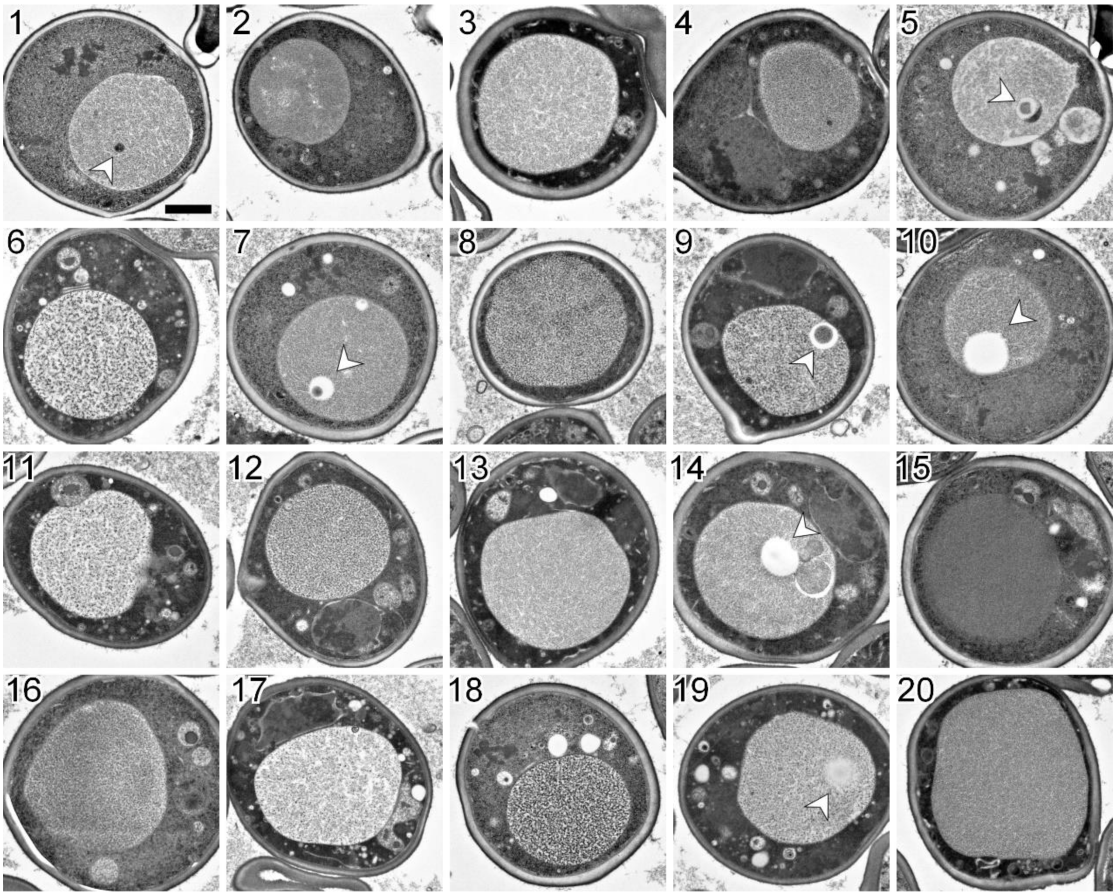

Supplementary Figure 6.

Electron microscopic analysis *vps23*Δ cells grown at 40.5°C for 6 h

Twenty cells are shown. Cells which appeared to be dying were not chosen. Arrow-heads indicate unidentified vesicle-like structures. Scale bar, 1 μm.

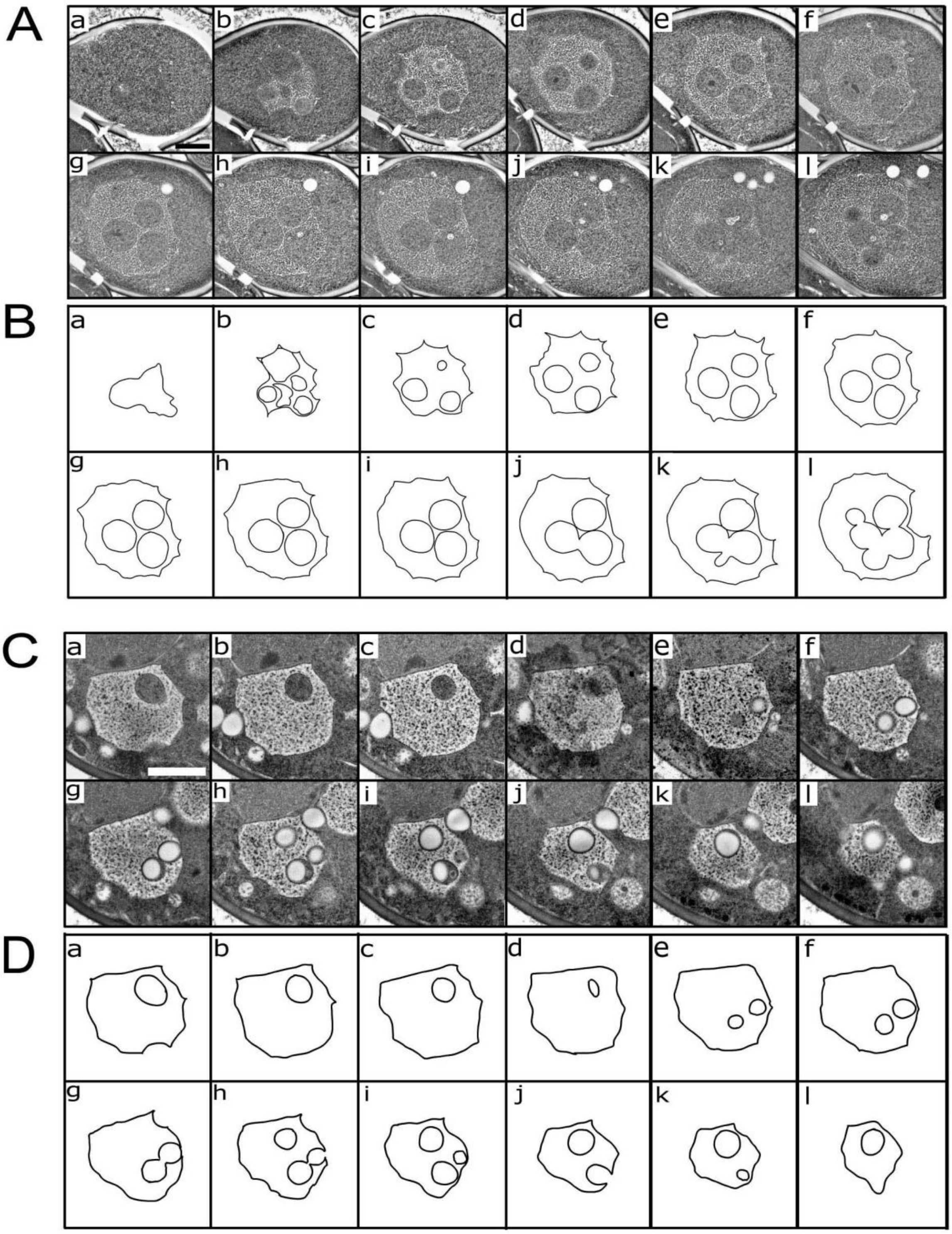

Supplementary Figure 7.

Serial sections of electron micrographs

Electron micrographs (A and C) and their traces (B and D) generated from 80-nm-thin serial sections of wild-type cells grown at 40.5°C for 6 h. Scale bar, 1 um.

Supplementary Video 1. Three-dimensional reconstructions of EM images in Fig. 6a

Supplementary Video 2. Three-dimensional reconstructions of traces of EM images in Fig. 6b

Supplementary Video 3. Flip images of Fig. 6a

Supplementary Video 4. Three-dimensional reconstructions of EM images in Fig. 6c

Supplementary Video 5. Three-dimensional reconstructions of traces of EM images in Fig. 6d

Supplementary Video 6. Flip images of Fig. 6c

Name	Genotype	Source/Reference
W303a	MATa *ade2-1 can1-100 his3-12,16 leu2-3,112 trp1-1 ura3-1*	Rothstein
W303α	MATα *ade2-1 can1-100 his3-12,16 leu2-3,112 trp1-1 ura3-1*	Rothstein
Y914	W303a *ubi4Δ::KanMX*	This study
US256	W303a *RPL24A-GFP::his3MX*	Ushimaru
Y1309	W303a *RPL24A-GFP::his3MX, pep4Δ::KanMX*	This study
Y1323	W303a *atg1Δ::KanMX*	This study
Y1273	W303a *atg9Δ::KanMX*	Ushimaru
Y995	W303a *vps23Δ::KanMX*	This study
Y1287	W303a *vps24Δ::KanMX*	This study
Y1375	W303a *ivy1Δ::KanMX*	This study
ScHY-2445	SEY6210 MATα *VPH1-GFPny::KanMX*	Ohsumi
Y1285	SEY6210 MATα *VPH1-GFPny::Hyg*	This study
Y1288	SEY6210 MATα *VPH1-GFPny::Hyg ubi4Δ::KanMX*	This study
Y1290	SEY6210 MATα *VPH1-GFPny:Hyg, Δvps23::KanMX*	This study
Y1320	SEY6210 MATα *VPH1-GFPny::KanMX, pep4Δ::HIS3*	This study
Y1229	W303a *LYP1-3HA::TRP1*	This study
Y1231	W303a *LYP1-3HA::TRP1, ubi4Δ::KanMX*	This study
Y1278	W303a *HXT3-3HA::TRP1*	This study
Y1280	W303a *HXT3-3HA::TRP1, ubi4Δ::KanMX*	This study
BY20695	MATα *his3Δ1, leu2Δ0, met15&Delta0ura3Δ0*	NBRP
Y1131	BY20695 *ubi4Δ::KanMX*	This study
BY4741	MATa *his3Δ1, leu2Δ0, met15Δ0, ura3Δ0*	Euroscarf
pep12	BY4741 *pep12Δ::KanMX4*	Euroscarf

Supplementary Table 1
Strains used in this study
