# Supplementary figures and images for: Accelerated invagination of vacuoles as a stress response in chronically heat-stressed yeasts
Source: Sci Rep. 2018 Feb 8;8:2644. doi: 10.1038/s41598-018-20781-8 (PMC5805771; doi:10.1038/s41598-018-20781-8)

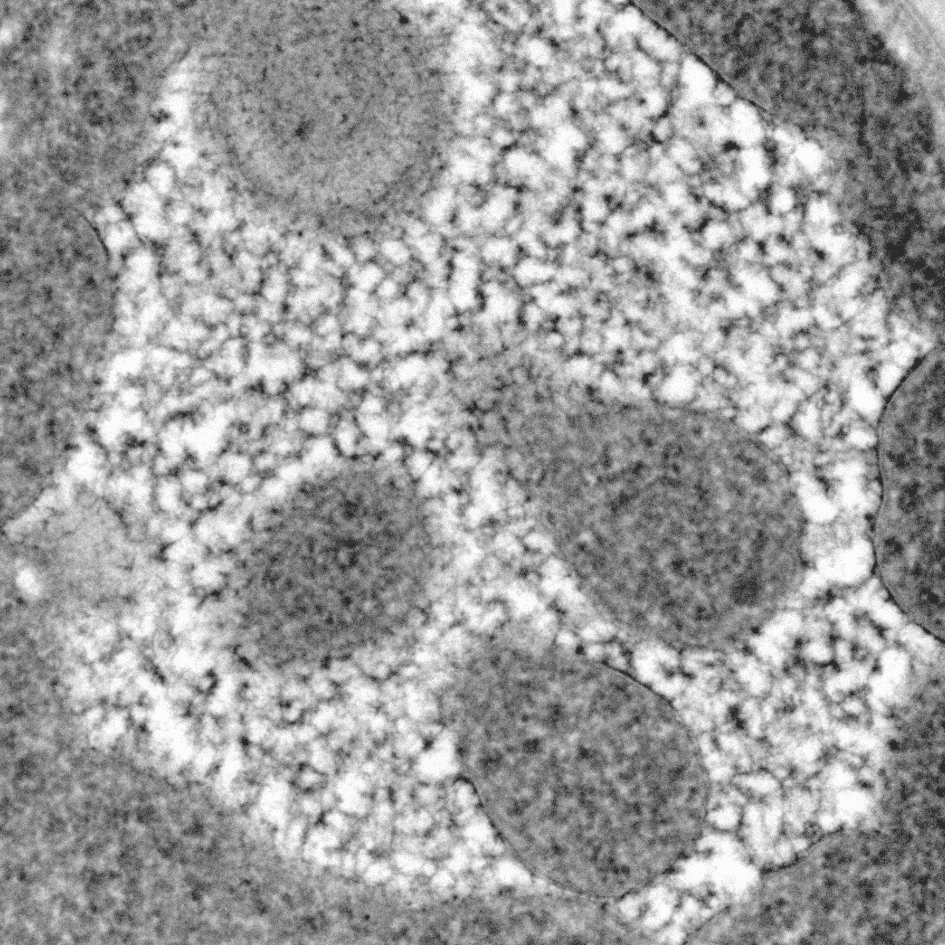

Supplement: Supplementary file 4 — Supplementary Video 3 [file 41598_2018_20781_MOESM4_ESM.gif]

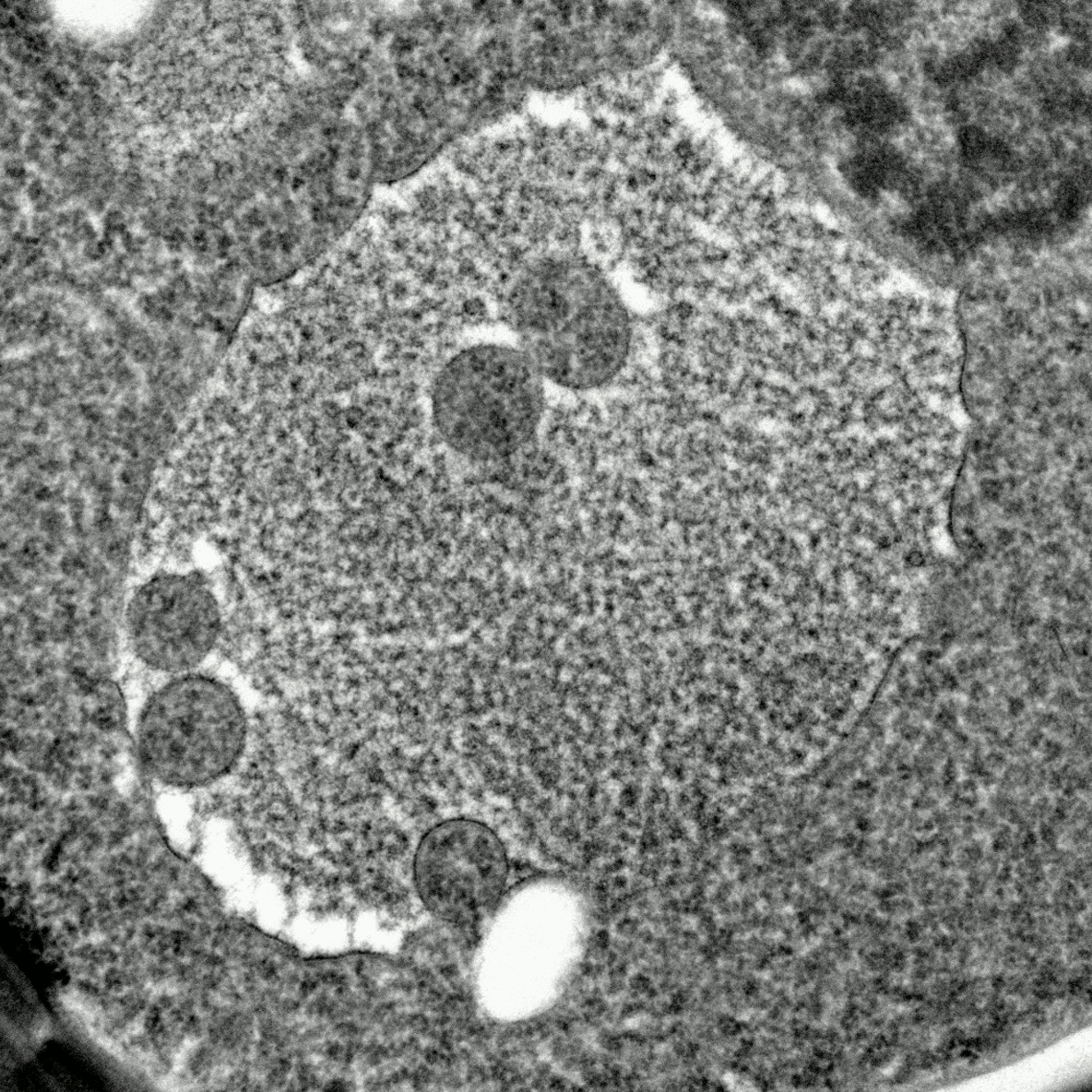

Supplement: Supplementary file 7 — Supplementary Video 6 [file 41598_2018_20781_MOESM7_ESM.gif]
